# Identifying client characteristics to predict homecare use more accurately: a Delphi-study involving nurses and homecare purchasing specialists

**DOI:** 10.1186/s12913-022-07733-9

**Published:** 2022-03-25

**Authors:** Anne O. E. van den Bulck, Arianne M. J. Elissen, Silke F. Metzelthin, Maud H. de Korte, Gertjan S. Verhoeven, Teuntje A. T. de Witte-Breure, Lieuwe C. van der Weij, Misja C. Mikkers, Dirk Ruwaard

**Affiliations:** 1 Faculty of Health, Medicine and Life Sciences, Department of Health Services Research, Maastricht University, Care and Public Health Research Institute (CAPHRI), P.O. Box 616, 6200 MD Maastricht, The Netherlands; 2 Department of Economics, Tilburg University, P.O. Box 90153, 5037 AB Tilburg, The Netherlands; 3 Dutch Healthcare Authority (NZa), P.O. Box 3017, 3502 GA Utrecht, The Netherlands; 4 Tilburg Law and Economics Center (TILEC), Tilburg University, P.O. Box 90153, 5000 LE Tilburg, The Netherlands

**Keywords:** Casemix, Client characteristics, Home care services, Classification, Prospective payment system, Policy

## Abstract

**Background:**

Case-mix based prospective payment of homecare is being implemented in several countries to work towards more efficient and client-centred homecare. However, existing models can only explain a limited part of variance in homecare use, due to their reliance on health- and function-related client data. It is unclear which predictors could improve predictive power of existing case-mix models. The aim of this study was therefore to identify relevant predictors of homecare use by utilizing the expertise of district nurses and health insurers.

**Methods:**

We conducted a two-round Delphi-study according to the RAND/UCLA Appropriateness Method. In the first round, participants assessed the relevance of eleven client characteristics that are commonly included in existing case-mix models for predicting homecare use, using a 9-Point Likert scale. Furthermore, participants were also allowed to suggest missing characteristics that they considered relevant. These items were grouped and a selection of the most relevant items was made. In the second round, after an expert panel meeting, participants re-assessed relevance of pre-existing characteristics that were assessed uncertain and of eleven suggested client characteristics. In both rounds, median and inter-quartile ranges were calculated to determine relevance.

**Results:**

Twenty-two participants (16 district nurses and 6 insurers) suggested 53 unique client characteristics (grouped from 142 characteristics initially). In the second round, relevance of the client characteristics was assessed by 12 nurses and 5 health insurers. Of a total of 22 characteristics, 10 client characteristics were assessed as being relevant and 12 as uncertain. None was found irrelevant for predicting homecare use. Most of the client characteristics from the category ‘Daily functioning’ were assessed as uncertain. Client characteristics in other categories – i.e. ‘Physical health status’, ‘Mental health status and behaviour’, ‘Health literacy’, ‘Social environment and network’, and ‘Other’ – were more frequently considered relevant.

**Conclusion:**

According to district nurses and health insurers, homecare use could be predicted better by including other more holistic predictors in case-mix classification, such as on mental functioning and social network. The challenge remains, however, to operationalize the new characteristics and keep stakeholders on board when developing and implementing case-mix classification for homecare prospective payment.

**Supplementary Information:**

The online version contains supplementary material available at 10.1186/s12913-022-07733-9.

## Background

Case-mix classification has been developed (and in some countries also implemented) as part of prospective payments in homecare, with the aim of making homecare more efficient and client-centred [1, 2]. Under case-mix classification, clients are allocated into groups that are relatively homogenous in their use of resources. Examples of case-mix models are the Home and Community Services Support Case-Mix (HCSS CM) model in New Zealand, which is based on the International Resident Assessment Instrument for Homecare (InterRAI-HC) data [1]. Most recently, in the Netherlands a case-mix model has been developed for Dutch homecare, based on Case-Mix Short Form (CM-SF) questionnaire data (Van den Bulck AOE, Elissen AMJ, Metzelthin SF, de Korte MH, Verhoeven GS, Mikkers MC, Ruwaard D. The Case-Mix Short-Form questionnaire for prospective payment of homecare services: Development and psychometric testing, Under review) [3].

To date one systematic literature review has been conducted that gathered knowledge on existing case-mix models for homecare and relevant predictors. This systematic literature review from Van den Bulck et al. (2020) found that existing homecare case-mix models focus largely on data on the client’s health (e.g. cognitive functioning and continence) and daily functioning (e.g. independence in washing and dressing) to predict homecare use [4]. However, based on these most common type of predictors, homecare case-mix models are only able to explain variance in homecare use to a limited extent (i.e. between 14 and 21% for newly developed models) [4]. Including other types of predictors could potentially improve the predictive value of case-mix models in homecare [3]. In a more recent study on predictors of homecare use, it was described that people in need for homecare are generally older, visit the general practitioner more often, and use more and/or expensive medications and aid devices [5]. Besides looking at the client’s health and daily functioning, homecare professionals apply a more holistic view of the client to accurately predict their need for homecare [1, 6, 7]. For example, according to the definition of Positive Health, health is more than simply the absence of disease, and client characteristics such as a client’s well-being and social functioning also affect health [8], and consequently also that client’s use of care. Looking beyond commonly included types of predictors may therefore be necessary in order to reduce unexplained variance in the predicted homecare use (Van den Bulck AOE, Elissen AMJ, Metzelthin SF, de Korte MH, Verhoeven GS, Mikkers MC, Ruwaard D. The Case-Mix Short-Form questionnaire for prospective payment of homecare services: Development and psychometric testing, Under review).

To establish a more holistic view of the client and thereby improve predictive value of homecare case-mix models, more insight is needed regarding which client characteristics should be included in case-mix models. There is a large number of possible predictors to include [4]. Therefore, it is valuable to involve district nurses and health insurers in the decision making process as they have experiential expertise and knowledge [9] on client characteristics that could predict homecare use. Involving nurses and insurers could also improve the model’s clinical relevance, and increase levels of professional support when implementing case-mix based prospective payments [1]. The aim of our study was therefore to evaluate which relevant predictors of homecare use are promising, or potentially even more relevant compared to the predictors that are currently commonly used, according to nurses and insurers.

## Methods

### Design

We conducted a two-round Delphi-study according to the RAND/UCLA Appropriateness Method (RAM) [9]. The aim of the RAM is to detect agreement between experts, rather than to reach consensus among them [9], which is in line with our study aim. Furthermore, the recommendations for Conducting and Reporting of Delphi Studies (CREDES) were followed to enhance the robustness of our study [10]. According to Dutch law on Medical Research (Human Subjects) Act (WMO), this study needed no ethical approval since the target group is not a vulnerable group, data is collected and processed anonymously, and participation was voluntary.

**Fig. 1:**
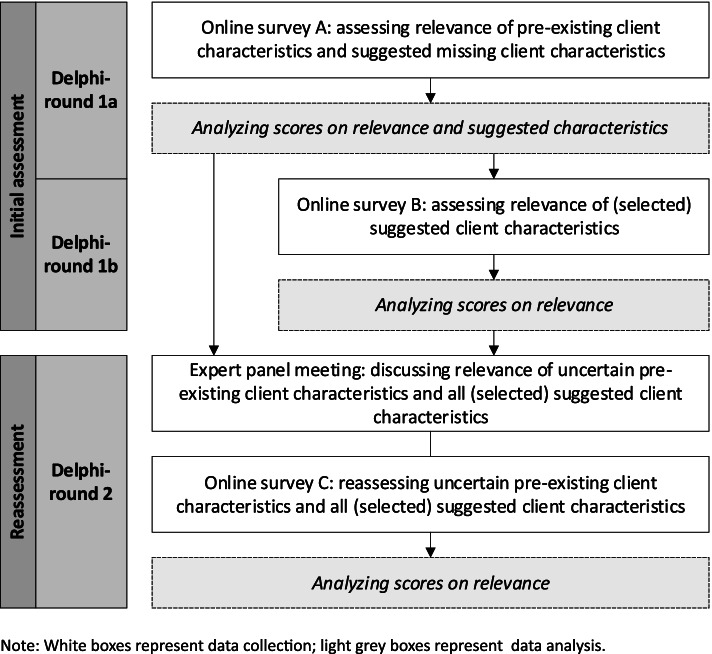
Steps in data collection and data analysis for the first and second Delphi-rounds

The following steps were conducted: the expert panel was selected; the first Delphi-round involving two online surveys (A and B) and the second Delphi-round with an expert panel meeting and an online survey (C) were prepared and carried out; and the survey data was analysed. Figure 1 provides an overview of the steps involving data collection and analysis in the two Delphi-rounds.

### Participants

District nurses and health insurers were selected as experts - i.e. people with significant knowledge of client characteristics that could be predictors of homecare use [11] - to participate in our study. When conducting a Delphi-study, it is advisable to include experts from diverse practice settings and diverse geographic settings [9]. Our aim was to involve a minimum of seven and a maximum of 15 participants per group [9].

District nurses are considered experts due to their experience in professional practice: they assess care needs of homecare clients based on a standard needs assessment and can fulfil a central role in the coordination of care from homecare clients. Therefore, they represent expertise in the area of nursing care, geriatric care and primary care. To recruit nurses for our study, we approached six Dutch homecare organizations who had previously participated in a pilot-study on the development of a case-mix model for prospective homecare payments. Those homecare organizations are located in different regions in the Netherlands. Each provider selected two or three district nurses from their organization. Three representatives from the Dutch Nurses Association (V&VN), who are also district nurses from diverse homecare organizations, were also asked to participate.

Health insurers are considered experts because of their experience in contracting homecare services, either as a homecare purchaser or as a policy adviser working for a health insurance company (both are considered homecare purchasing specialists). Therefore, they represent expertise in the area of health policy and health economics. The aim was to at least include experts from the four health insurance companies with the largest market share in the Netherlands, which together represent 85% of the market [12]. The homecare organizations were asked to propose homecare purchasers and/or policy advisers from the health insurance companies which they had the most frequent contact with regarding contracting homecare services. Additionally, the remaining six health insurance companies in the Netherlands with a smaller market share (i.e. between 1 and 4%) were asked to participate.

An e-mail was sent to the proposed participants providing information on the aim of the study, its design and the inclusion criteria for experts. Participants who wished to take part in our study were asked to indicate their availability so that the expert panel meeting could be scheduled. Additionally, informed consent was asked from the participants. If a participant did not believe they had the right knowledge on the subject or did not want to participate, they were asked to suggest a colleague instead.

### First Delphi-round

The first Delphi-round consisted of two online surveys – A and B – using the survey tool Qualtrics [13].

#### Data collection

Online survey A started with an informed consent declaration, and a list defining the terms used in the survey. Participants were asked to provide background information including their name, sex, age, education, organization, and current job title. Their names were only used to inform the participants of their own scores, and so that the moderator would have an overview of the scores of the participants in the expert panel meeting (as prescribed by the RAM [9]). Other than that, all data collected was fully anonymized by removing the names from the data.

The participants were then asked to assess the relevance of client characteristics for predicting homecare use. The pre-existing client characteristics that had to be assessed were selected from our previously developed Case-Mix Short Form (CM-SF) questionnaire (Van den Bulck AOE, Elissen AMJ, Metzelthin SF, de Korte MH, Verhoeven GS, Mikkers MC, Ruwaard D. The Case-Mix Short-Form questionnaire for prospective payment of homecare services: Development and psychometric testing, Under review). The CM-SF questionnaire was developed to collect data for homecare case-mix classification for prospective payment, independently of the nursing classification system used. Using this 11-item questionnaire, data are collected on the most common predictors of homecare use in existing case-mix models [4, 6]. It assesses a homecare client’s current functioning with regard to 11 client characteristics: 1) Illness prognosis, 2) Meal preparation, 3) Eating and drinking, 4) Continence, 5) Toileting, 6) Mobility, 7) Dressing, 8) Washing/showering, 9) Medication use, 10) Cognitive skills for daily decision making, and 11) Informal care. All 11 characteristics in the CM-SF were included in our Delphi-survey. To help the participants reflect on potentially relevant predictors of homecare use, we divided the survey into six categories: 1) Daily functioning, including eight CM-SF questionnaire items: meal preparation, eating and drinking, continence, toileting, mobility, dressing, washing/showering, and medication use; 2) Physical health status; 3) Mental health status and behaviour, including one CM-SF questionnaire item: cognitive skills for daily decision making; 4) Health skills; 5) Social environment and network, including one CM-SF questionnaire item: informal care; and 6) Other, including one CM-SF questionnaire item: Illness prognosis.

The relevance of the 11 pre-existing characteristics was scored by the participants using a 9-Point Likert scale, ranging from 1 (completely irrelevant) to 9 (extremely relevant). If the participants found that one or more relevant client characteristics was missing, they could add these client characteristics (up to a maximum of six per category). For each client characteristic suggested, participants were asked to provide a brief definition and, where applicable, refer to an existing question or questionnaire to measure it objectively. An example of the survey questions (translated from Dutch to English) is provided in Additional file 1.

All participants who agreed to take part were sent the link for survey A by e-mail. The participants had ten days to complete the survey, starting on 10 March 2021. Two reminders were sent to increase the response rate.

For online survey B, conducted prior to the discussion of the expert panel meeting, participants assessed the relevance of a selection of the suggested client characteristics in survey A. This was to encourage the participants think about an initial score for all the characteristics before the discussion. The suggested characteristics were assessed in the same way as in survey A – i.e. by scoring their relevance on a 9-Point Likert scale.

#### Data analysis

We used descriptive statistics to analyse the background characteristics of the participants (i.e. frequencies, percentages, and means). Analyses of the relevance of each client characteristic were guided by previous studies in which the relevance of client characteristics was assessed [6, 13–15]. For both surveys, we used median scores to determine relevance: client characteristics with a median between 1 and 3 were interpreted as irrelevant, a median between 4 and 6 as uncertain, and a median between 7 and 9 as relevant. Furthermore, inter-quartile ranges (IQR) were used to determine the level of consensus between participants: an IQR ≤ 2 was considered as sufficient consensus and IQR > 2 as a lack of consensus. The combination of the median and IQR determined how the relevance of each client characteristic was judged. A client characteristic was considered relevant if it had a median between 7 and 9, combined with an IQR ≤ 2; irrelevant if it had a median between 1 and 3, combined with an IQR ≤ 2; and uncertain if it had a median between 4 and 6, or IQR > 2. Sensitivity analyses were performed to check for differences between nurses and insurers regarding the relevance assigned. The results of survey A were analysed prior to survey B and the second Delphi-round.

We performed content analysis [16] to analyse the client characteristics suggested by the participants in survey A. One researcher reordered each of the characteristics by grouping together similar suggestions and defining these based on the definitions provided by the participants. If too many characteristics were mentioned to assess and discuss during the expert panel meeting, the researchers selected the potentially most relevant suggestions. The researchers involved in this selection have expertise in the areas of (home care) nursing, primary care, health policy and health economics. We selected characteristics that: 1) were known predictors of homecare use in the literature; 2) involved a predictor category that had not yet been included in the CM-SF questionnaire; or 3) were identified as lacking in the CM-SF questionnaire by (among others) district nurses in the pilot-study [3, 17]. Suggestions that overlapped with items already in the CM-SF questionnaire or for which no definition was provided were excluded. The researchers discussed this until agreement was reached regarding the selection.

### Second Delphi-round

#### Data collection

The second Delphi-round consisted of an expert panel meeting and online survey C. Due to the large difference in perspective between district nurses and insurers, and the potential barriers to speaking openly, we decided to hold two separate expert panel meetings: one for district nurses, and one for insurers. Each two-hour expert panel meeting was held online using Zoom video-conferencing software. The meeting was recorded using an external voice recorder. All participants who completed the first Delphi-round survey were invited to participate. One researcher chaired the meeting and moderated the discussion, one researcher timed the meeting and was able to ask questions, and one researcher (i.e. a panel observer) took notes. In advance of the meeting, the participants were sent a document revealing their individual scores, the median and range of the group scores of the first Delphi-round. The moderator also had a personalized score sheet showing the scores of each participant for each client characteristic.

During the meeting, the participants shared their thoughts and discussed their thinking regarding the scores they had given to each client characteristic. Pre-existing client characteristics that were found to be consensually relevant or irrelevant in survey A in the first Delphi-round were not discussed.

At the end of the meeting, the participants completed survey C in which they reassessed a) pre-existing client characteristics that had initially been found to be uncertain, and b) all (selected) suggested client characteristics (because no results on relevance for all participants were available yet). Reassessment was carried out in the same way as the initial assessment in survey A and B, i.e. by scoring relevance on a 9-Point Likert scale. If the participant’s score did not change between rounds, they could fill in the same score. Unlike in survey A, it was not possible to suggest new client characteristics in this survey.

#### Data analysis

The scores were analysed in the same way as in survey A and B, i.e. by determining median and IQR.

## Results

### Background characteristics of participants

Table 1 presents an overview of (the background characteristics of) the participating experts. All 16 contacted nurses agreed to participate and filled in survey A in the first Delphi-round (100%). Of these, 12 nurses (75%) also participated in the expert panel meeting and completed surveys B and C. Almost all the participating nurses were district nurses working at a homecare organization. Six out of eight contacted insurers agreed to participate and filled in survey A (75%). The two insurers who did not participate were already being represented by colleagues from their health insurance company who had agreed to participate. In the second Delphi-round, five insurers were able to participate in the expert panel meeting and surveys B and C (63%). Most participating insurers worked as homecare purchasers. Reasons given for not participating in the second Delphi-round (for both nurses and insurers) were lack of time, other appointments, or maternity leave.

**Table 1:** Background characteristics of the participants (per Delphi-round and per Delphi-group)

	**Delphi-round 1a**			**Delphi-round 1b and 2**		
	Total	Nurses	Insurers	Total	Nurses	Insurers
	*N* = 22	*n* = 16	*n* = 6	*N* = 17	*n* = 12	*n* = 5
**Gender (n)**						
Male	5	1	4	2	0	2
Female	17	15	2	15	12	3
**Age (range, average)**	24–65 (39)	24–65 (36)	31–61 (48)	24–61 (35)	24–49 (32)	31–61 (41)
**Education (n)**						
University of Applied Science	16	15	1	12	11	1
University	6	1	5	5	1	4
**Organization (n)**^**a**^						
Homecare organization	15	15	0	11	11	0
Dutch Nurses Association	3	3	0	2	2	0
Health insurance company	6	0	6	5	0	5
**Job title (n)**^**a**^						
District nurse	14	14	0	11	11	0
Homecare purchaser	5	0	5	3	0	3
Policy advisor insurer	0	0	1	1	0	1
Other^b^	3	2	1	2	1	1

^a^Some participants were working at multiple organizations or held multiple positions. Frequencies therefore do not add to N

^b^Process director electronic health records at homecare organization, policy advisor at homecare organization (only Delphi-round 1), policy manager at health insurance company

### First Delphi-round

The participants assessed the relevance of 11 pre-existing client characteristics. The results on the relevance of each characteristic are presented in Table 2. In total, three client characteristics (27%) were considered relevant (median 7–9 and IQR ≤ 2); these were ‘Washing/showering’, ‘Cognitive skills for daily decision making’, and ‘Illness prognosis’. The relevance of the other eight client characteristics (73%) was found to be uncertain (median 4–6 or IQR > 2), mainly due to a lack of consensus between participants (i.e. IQR > 2). None of the characteristics was considered irrelevant as a predictor of homecare use.

**Table 2:** Results on client characteristics’ relevance (median, IQR) per Delphi-round, sorted by category of client characteristics

	**Delphi round 1a and 1b**				**Delphi round 2**			
	Median	Q1-Q3	IQR	Judgment	Median	Q1-Q3	IQR	Judgment
**Daily functioning**								
Meal preparation	6	2.75–7.0	4.25	Uncertain	5	2.5–7.0	4.50	Uncertain
Eating and drinking	7	5.75–8.0	2.25	Uncertain	7	5.0–7.0	2.00	Relevant
Continence	6	4.5–7.0	2.50	Uncertain	5	3.0–6.5	3.50	Uncertain
Toileting	7	3.75–8.25	4.50	Uncertain	7	4.0–8.0	4.00	Uncertain
Mobility	7	5.0–9.0	4.00	Uncertain	7	5.0–7.5	2.50	Uncertain
Dressing	7	5.0–8.0	3.00	Uncertain	6	5.0–7.5	2.50	Uncertain
Washing/showering	7	5.0–7.0	2.00	Relevant	-	-	-	-
Medication use	7	4.75–8.0	3.25	Uncertain	7	5.0–8.0	3.00	Uncertain
**Physical health status**								
Multi-morbidity^a^	7	6.5–7.5	1.00	Relevant	7	7.0–7.0	0.00	Relevant
Skin problems^a^	7	5.0–8.0	3.00	Uncertain	7	5.0–8.0	3.00	Uncertain
Vision and hearing^a^	5	3.5–6.0	2.50	Uncertain	5	3.0–6.0	3.00	Uncertain
Malnutrition^a^	6	4.5–6.0	1.50	Uncertain	6	5.0–6.5	1.50	Uncertain
**Mental health status and behaviour**								
Cognitive skills for daily decision making	8	7.0–9.0	2.00	Relevant	-	-	-	-
Mental functioning^a^	7	6.0–8.0	2.00	Relevant	7	6.0–8.0	2.00	Relevant
Resilience^a^	7	6.5–7.5	1.00	Relevant	7	6.5–8.0	1.50	Relevant
Dementia^a^	7	6.5–8.0	1.50	Relevant	5	3.0–7.5	4.50	Uncertain
Self-management and self-direction^a^	7	6.0–8.5	2.50	Uncertain	8	6.5–9.0	2.50	Uncertain
**Health literacy**								
Learning ability^a^	7	6.0–8.5	2.50	Uncertain	8	7.0–8.0	1.00	Relevant
**Social environment and network**								
Informal care	8	6.0–9.0	3.00	Uncertain	9	6.5–9.0	2.50	Uncertain
Social network^a^	7	7.0–8.0	1.00	Relevant	8	7.0–8.5	1.50	Relevant
**Other**								
Illness prognosis	8	7.0–9.0	2.00	Relevant	-	-	-	-
Need for technical nursing care^a^	6	5.5–8.0	2.50	Uncertain	7	6.0–8.0	2.00	Relevant

Note: Pre-existing client characteristics that were assessed as relevant in the first Delphi-round were not re-assessed in the second Delphi-round

^a^Characteristics were selected from the client characteristics suggested by the participants in survey A. These were assessed in survey B (in Delphi-round 1b) and re-assessed in survey C

In the open-ended questions in survey A, participants suggested 142 client potentially relevant characteristics for predicting homecare use. After these were grouped, we ended up with 53 unique client characteristics, that were added to a corresponding predictor category (see Additional file 2). On average, nine client characteristics were added to each category, ranging from four in the ‘Social environment and network’ category to 14 in the ‘Others’ category. Of the 53 client characteristics, the 11 potentially most relevant were selected: ‘Multi-morbidity’, ‘Skin problems’, ‘Vision and hearing’, ‘Malnutrition’, ‘Mental functioning’, ‘Resilience’, ‘Dementia’, ‘Self-management and self-direction’, ‘Learning ability’, ‘Social network’, and ‘Need for technical nursing care’.

The results regarding the relevance of each of the 11 suggested client characteristics are shown in Table 2 (marked with an *). Five characteristics (45%) were assessed as relevant (median 7–9 and IQR ≤ 2). The relevance of the remaining six characteristics (55%) was uncertain (median 4–6 or IQR > 2), due to a lack of consensus (i.e. IQR > 2) and/or a low median score (median 4–6). Again, none of the characteristics was considered irrelevant as a predictor of homecare use.

According to the sensitivity analyses (see Additional file 3), the nurses seem to have given the client characteristics higher median scores than the insurers. Additionally, there was more consensus regarding relevance (i.e. a relatively lower IQR) among the nurses than among the insurers.

### Second Delphi-round

After the discussion during the expert panel meeting, the participants reassessed the relevance of the eight pre-existing client characteristics that were found to be uncertain (see Table 2). With the exception of ‘Eating and drinking’, on which there was consensus regarding relevance following reassessment, the seven other pre-existing client characteristics that were reassessed remained uncertain. Of the client characteristics that had been suggested, the characteristics ‘Learning ability’ and ‘Need for technical nursing care’ were found to be relevant after reassessment, while ‘Dementia’ shifted from relevant to uncertain.

After the second Delphi-round, there was thus agreement between participants on the relevance of 10 of the 22 client characteristics for predicting homecare use. Overall, more of the client characteristics that had been suggested were considered relevant than the pre-existing characteristics (6/11 vs. 4/11, respectively). Furthermore, there were differences in the number of client characteristics in each predictor category that were assessed as relevant (see Table 2).

In the results of the sensitivity analysis (see Additional file 3), no clear changes were found in the medians (i.e. some increased and others decreased) or the consensus (i.e. on some characteristics more consensus, and on others less consensus was found) in the reassessment by the nurses compared to the reassessment by the insurers. Furthermore, compared to the assessment of all the participants combined in the second round, the nurses’ final assessment of relevance deviated on two client characteristics (i.e. one was relevant instead of uncertain; one was uncertain instead of relevant). For the insurers, the assessment deviated on seven client characteristics (mainly less relevant compared to all participants).

## Discussion

In this Delphi-study, district nurses and homecare insurers discussed and assessed the relevance of various client characteristics as predictors of homecare use. Eleven pre-existing characteristics from the CM-SF questionnaire were assessed. The participants also suggested 142 client characteristics as potentially relevant predictors of homecare use: we were able to group these suggested characteristics into 53 unique characteristics and, after discussion, we selected 11 for expert assessment. The relevance of the client characteristics in the category of ‘Daily functioning’ was mainly assessed as uncertain, except for ‘Eating and drinking’ and ‘Washing/showering’. Client characteristics from other categories were more likely to be considered relevant: ‘Multi-morbidity’ (from the category ‘Physical health status’), ‘Cognitive skills for daily decision making’, ‘Mental functioning’, and ‘Resilience’ (from the category ‘Mental health status and behaviour’), ‘Learning ability’ (from the category ‘Health literacy’), ‘Social network’ (from the category ‘Social environment and network’), and ‘Illness prognosis’ and ‘Need for technical nursing care’ (from the category ‘Other’). In total, 10 client characteristics were assessed as relevant and 12 as uncertain. The participants did not consider any of the characteristics as irrelevant for predicting homecare use.

The participants’ view on which characteristics are relevant predictors of homecare use deviates from the set of characteristics currently included in existing case-mix models for homecare. In a systematic literature review from Van den Bulck et al. (2020), we found that characteristics from the ‘Daily functioning’ category were included in all existing case-mix models [4]. Notably, these characteristics were mainly assessed as of uncertain relevance by our participants. Examples include ‘Toileting’, ‘Mobility’, and ‘Dressing’. At the same time, the majority of characteristics that were assessed as relevant by nurses and insurers, such as ‘Resilience’, ‘Learning ability’, and ‘Social network’, are seldom included in existing case-mix models [4]. One possible explanation relates to the difficulty of operationalizing these characteristics in a concise and standardized manner. For example, existing questionnaires relating to the ‘Social network’ characteristic include numerous sub-items and multiple aspects – e.g. the number of social contacts that a client has, what kind of social contact a client has, or whether a client is satisfied with his/her own social network (Van den Bulck AOE, Elissen AMJ, Metzelthin SF, de Korte MH, Verhoeven GS, Mikkers MC, Ruwaard D. The Case-Mix Short-Form questionnaire for prospective payment of homecare services: Development and psychometric testing, Under review). In addition to this, these client characteristics are difficult to assess. For example, it can be challenging to assess the client’s resilience or social network, because it requires good an probably long-term knowledge of the client. Another possible explanation for this relates to the explanation for a client’s care needs. Characteristics in the category ‘Daily functioning’ are more ‘downstream’ (i.e. proximal) characteristics that influence a client’s homecare use more directly [18]. By contrast, most of the suggested characteristics assessed as relevant are more ‘upstream’ (i.e. distal) characteristics, which are fundamental causes of a client’s homecare use and that may have an influence on one or multiple downstream characteristics [18]. For example, having few social contacts (an upstream characteristic) may not necessarily be a direct reason for receiving homecare, but when combined with dementia (a downstream characteristic) it may cause the client to have a (higher) need for homecare. The associations between several characteristics and homecare use have also been demonstrated in other studies. For example, for ‘Multi-morbidity’, homecare use appears to increase with the number of chronic diseases that a client has [19]; and with regard to ‘Mental functioning’, homecare use is higher for clients with depressive symptoms [20] and clients with dementia [21] compared to those without.

The development of a case-mix classification is affected by the tension between the need for a relatively simple model and the broad range of views on homecare policy and practice. The participants suggested a large number of additional unique client characteristics (more than 50) as potentially relevant predictors of homecare use. One possible explanation for this would be the broad perspective on homecare among the participants, who have experienced a great variety of increasingly complex homecare clients and interventions. This broad perspective might be difficult to reconcile with the need for relatively simple CM-SF questionnaire items. With regard to homecare policy, the Dutch government is focusing on encouraging clients to live independently at home for as long as possible by adopting approaches such as Positive Health [8] and “reablement” (i.e. “a person-centred, holistic approach that aims to enhance an individual's physical and/or other functioning, to increase or maintain their independence in meaningful activities of daily living at their place of residence and to reduce their need for long-term services”) [22]. Driven by national-level policies [23, 24], but also developments at the international level [25, 26], nurses and insurers are increasingly striving to improve the independence and self-reliance of clients. This focus within homecare policy can thus also be expected to show through in how nurses and insurers view client characteristics when seeking to predict homecare use (i.e. by suggesting additional characteristics such as ‘Self-management and self-direction’). What is more, when district nurses assess a client’s homecare needs, they not only determine functional limitations, such as difficulties with dressing, but they look specifically for the etiology that lies behind it, such as a client’s resilience or learning ability [27]. However, the goal of the CM-SF questionnaire and of a case-mix model is not to *explain* homecare, but to *predict* homecare use adequately, and this goal may deviate from or be narrower than the broad focus of policy and the views of experts within the homecare sector.

One strength of this study is its robustness, enhanced by its compliance with the RAM and CREDES guidelines when performing and reporting on our study. Furthermore, we included two different groups of experts in the field of homecare: nurses and insurers. On the one hand, discussions of the relevance of the characteristics were held separately for each group, so that all participants would feel comfortable enough to share their views. On the other hand, the results of the assessments of both groups were combined, so that they had quantitative input from the other group to help them reflect on their own assessments. Another strength was the initial assessment of the relevance of suggested client characteristics prior to the expert panel meeting to ensure that all participants had the opportunity to consider their view before the discussion. A limitation of our study is that the researchers selected the 53 suggested client characteristics. It is unclear how the total group of experts would have rated the client characteristics that were omitted. However, since the selected characteristics were assessed as relevant relatively often, we may conclude that an appropriate selection was made. Another limitation is the small sample size of the participating insurers. This could have led to the relatively low consensus among this group compared to the nurses. However, since the participants represented four health insurers with a combined 85% of market share in the Netherlands, we assume that the lack of consensus and the scores provided are a relatively accurate representation of the views of Dutch health insures on homecare use predictors.

The participating nurses and insurers seem to agree that characteristics beyond the client’s health and daily functioning may be relevant for case-mix classification, and that a more holistic view of the client could be useful in predicting homecare use. For other countries that have been developing homecare case-mix classification, this knowledge could be used to improve their models. Moreover, our findings also guide future research on homecare case-mix classification, for example for countries that still are to develop certain models. However, the challenge remains determining which relevant suggested characteristics are suitable for case-mix classification due to the difficulty of operationalizing these characteristics. To continue the development of case-mix based prospective payment in the Netherlands, we would therefore recommend to conduct additional research with stakeholders in homecare – including district nurses, insurers, homecare providers, the nurses association – to discuss how the client characteristics assessed as relevant can best be operationalized and measured. Furthermore, to avoid misunderstandings (e.g. on why certain characteristics are or are not included as predictors for case-mix classification) and maintain professional support, it would be essential for policy makers to involve district nurses and insurers (and possibly other parties) in the development of the CM-SF questionnaire (for example) and when implementing case-mix classification for prospective payment. This is necessary because, according to our study, client characteristics that end up in case-mix classification may not necessarily be representative of homecare as a whole.

## Conclusions

While some client characteristics have proven their relevance as predictors of the use of homecare in existing homecare case-mix models, these models could still be improved further. In this Delphi-study, we have found that, according to district nurses and health insurers, it may be possible to achieve higher predictive value by including a more holistic view in the predictors in the case-mix model. However, the challenge remains keeping all stakeholders on board as their views on how case-mix classification should be formed and used may differ. New client characteristics namely still have to be operationalized (which is rather complex) and to prove their predictive value, and characteristics that could have high predictive value may not be in line with the full breadth of daily homecare practice.

## Supplementary Information

**Additional file 1.** Example of survey questions.

**Additional file 2. ** The 53 unique client characteristics that were suggested.

**Additional file 3. ** Sensitivity analysis.

## Data Availability

Data regarding the characteristics of the participants cannot be shared publicly because they may be traceable. All other data underlying the results presented in the study are available from a public repository at the Open Science Framework (OSF) via the following link: https://osf.io/zf94b/.

## Abbreviations

CREDES: Recommendations for Conducting and Reporting of Delphi Studies
CM-SF: Case-Mix Short Form
IQR: Inter-quartile range
RAM: RAND/UCLA Appropriateness Method
