# Supplementary material for: Identifying client characteristics to predict homecare use more accurately: a Delphi-study involving nurses and homecare purchasing specialists
Source: BMC Health Serv Res. 2022 Mar 25;22:394. doi: 10.1186/s12913-022-07733-9 (PMC8957197; doi:10.1186/s12913-022-07733-9)
Supplement: Supplementary file 1 — Additional file 1. Example of survey questions. [file 12913_2022_7733_MOESM1_ESM.pdf]

ADDITIONAL FILES

Additional file 1. Example of survey questions

Additional file 2. The 53 unique client characteristics that were suggested

Additional file 3. Sensitivity analysis regarding the relevance of client characteristics (median and IQR) per Delphi-round, comparing results for all participants, nurses, and insurers

Additional file 1. Example of survey questions

Category 1. Daily functioning

For example: (instrumental) activities of daily living (ADL and IADL).

From the category 'Daily functioning', the following client characteristics are included in the Case-Mix Short Form (CM-SF) questionnaire:

- Meal preparation
- Eating and drinking
- Continence
- Toileting
- Mobility
- Dressing
- Washing/showering
- Medication use

Meal preparation
Answer options in the CM-SF:

- *The client prepares all meals independently.*
- *The client needs some help from others (e.g. encouragement, supervision, or physical support) when preparing (a) meal(s).*
- *Meals need to be prepared fully by others.*

How relevant do you consider the characteristic of 'Meal preparation' to predicting the need for homecare?

Totally irrelevant											Extremely relevant
○	○	○	○	○	○	○	○	○	○	○	○

[…]

Do you think any additional client characteristic(s) from the category 'Daily functioning' is/are relevant to predicting the need for homecare?
If yes:

Client characteristic 1 (max. 6 words)	……………………………………………………………………
Definition client characteristic 1	…………………………………………………………………… ……………………………………………………………………
Suggest an existing question or questionnaire to objectively measure client characteristic 1 (optional)	…………………………………………………………………… …………………………………………………………………… ……………………………………………………………………

Additional file 2. The 53 unique client characteristics that were suggested

The 53 suggested client characteristics (per category of predictors) that resulted from grouping the 142 client characteristics suggested by the participants:

- Category 'Daily functioning' (11):
  1. Daily routine
  2. Use of telephone
  3. Housework
  4. Grocery shopping
  5. Sleeping
  6. Managing (financial) administration
  7. Taking the initiative
  8. ADL tasks*
  9. Stocking*
  10. Changing incontinence material*
  11. Medication*
- Category 'Physical health status' (13):
  12. Multimorbidity**
  13. Diabetes
  14. Skin problems**
  15. Airway functioning
  16. Vision and hearing**
  17. Pain
  18. Polypharmacy
  19. Risk of falls
  20. Physical fitness
  21. Malnutrition**
  22. Excess weight
  23. Mobility*
  24. Progressive disease*
- Category 'Mental health status and behaviour' (7):
  25. Mental functioning**
  26. Resistance to receiving care
  27. Resilience**
  28. Dementia**
  29. Communication skills
  30. Self-management and self-direction**
  31. Cognitive skills*
- Category 'Health literacy' (7):
  32. Health literacy
  33. Healthy lifestyle
  34. Learning ability**
  35. Compliance with therapy
  36. Digital skills
  37. Awareness of support options
  38. Capacity for self-care*
- Category 'Social environment and network' (4):
  39. Loneliness
  40. Social network**
  41. Participation in social activities
  42. Meaning
- Category 'Other' (11):
  43. Financial resources
  44. Formal care network
  45. Living in an urban area
  46. Need for technical nursing care**
  47. Quality of transfer from the hospital
  48. Quality of life
  49. Frailty
  50. Availability of facilities
  51. Care needs at multiple levels*
  52. Terminal status*
  53. (Illness) prognosis/Stability*

* Client characteristics that (partially) overlap with one or several of the 11 pre-existing client characteristics that were already included in the Case-Mix Short Form questionnaire.
** The 11 suggested client characteristics that were selected by the researchers for further assessment.

Additional file 3. Sensitivity analysis regarding the relevance of client characteristics (median and IQR) per Delphi-round, comparing results for all participants, nurses, and insurers

	**Delphi round 1**									**Delphi round 2**								
	Total			Nurses			Insurers			Total			Nurses			Insurers		
	Median	Q1-Q3	IQR	Median	Q1-Q3	IQR	Median	Q1-Q3	IQR	Median	Q1-Q3	IQR	Median	Q1-Q3	IQR	Median	Q1-Q3	IQR
Pre-existing																		
Meal preparation	6.00	2.75-7.0	4.25	6.00	5.0-7.0	2.00	4.00	1.75-6.25	4.50	5.00	2.5-7.0	4.50	6.00	4.25-7.0	2.75	3.00	2.0-4.0	2.00
Eating and drinking	7.00	5.75-8.0	2.25	7.00	6.0-8.75	2.75	6.00	4.75-8.0	3.25	7.00	5.0-7.0	2.00	7.00	7.0-7.75	0.75	5.00	3.5-6.0	2.50
Continence	6.00	4.5-7.0	2.50	6.00	5.0-7.0	2.00	5.50	3.0-6.5	3.50	5.00	3.0-6.5	3.50	5.50	3.25-7.0	3.75	5.00	3.0-5.5	2.50
Toileting	7.00	3.75-8.25	4.50	7.00	3.75-9.0	5.25	4.50	3.75-7.25	3.50	7.00	4.0-8.0	4.00	7.50	5.25-8.0	2.75	4.00	3.5-7.5	4.00
Mobility	7.00	5.0-9.0	4.00	7.50	5.25-9.0	3.75	6.50	4.75-7.5	2.75	7.00	5.0-7.5	2.50	7.00	5.0-8.0	3.00	5.00	4.5-7.0	2.50
Dressing	7.00	5.0-8.0	3.00	6.50	5.0-8.0	3.00	7.00	5.0-8.25	3.25	6.00	5.0-7.5	2.50	6.50	5.0-7.75	2.75	5.00	5.0-7.5	2.50
Washing	7.00	5.0-7.0	2.00	7.00	5.0-7.0	2.00	7.00	5.75-7.5	1.75	-	-	-	-	-	-	-	-	-
Medication use	7.00	4.75-8.0	3.25	7.50	6.25-8.75	2.50	4.50	2.75-7.25	4.50	7.00	5.0-8.0	3.00	7.00	6.25-8.75	2.50	5.00	3.5-6.0	2.50
Cognitive skills for daily decision making	8.00	7.0-9.0	2.00	8.50	7.25-9.0	1.75	7.00	3.75-9.0	5.25	-	-	-	-	-	-	-	-	-
Informal care	8.00	6.0-9.0	3.00	8.00	7.25-9.0	1.75	7.50	5.75-9.0	3.25	9.00	6.5-9.0	2.50	9.00	8.25-9.0	0.75	7.00	5.5-9.0	3.50
Illness prognosis	8.00	7.0-9.0	2.00	8.00	7.0-9.0	2.00	8.00	6.0-8.25	2.25	-	-	-	-	-	-	-	-	-
Suggested																		
Multimobidity	7.00	6.5-7.5	1.00	7.00	7.0-7.75	0.75	7.00	6.0-7.5	1.50	7.00	7.0-7.0	0.00	7.00	7.0-7.75	0.75	7.00	5.5-7.0	1.50
Skin problems	7.00	5.0-8.0	3.00	7.00	5.0-8.0	3.00	7.00	4.0-8.0	4.00	7.00	5.0-8.0	3.00	7.00	5.0-8.0	3.00	5.00	4.0-7.0	3.00
Vision and hearing	5.00	3.5-6.0	2.50	6.00	4.25-6.0	1.75	4.00	3.0-5.5	2.50	5.00	3.0-6.0	3.00	5.50	3.25-6.0	2.75	3.00	3.0-4.5	1.50
Malnutrition	6.00	4.5-6.0	1.50	6.00	4.25-6.75	2.50	5.00	4.5-5.5	1.00	6.00	5.0-6.5	1.50	6.00	5.25-7.75	2.50	5.00	3.5-5.0	1.50
Mental functioning	7.00	6.0-8.0	2.00	7.00	6.0-8.0	2.00	7.00	6.0-7.5	1.50	7.00	6.0-8.0	2.00	7.00	6.25-8.0	1.75	7.00	6.0-7.5	1.50
Resilience	7.00	6.5-7.5	1.00	7.00	6.25-8.0	1.75	7.00	6.5-7.0	0.50	7.00	6.5-8.0	1.50	8.00	6.25-8.0	1.75	7.00	6.5-7.5	1.00
Dementia	7.00	6.5-8.0	1.50	7.00	6.0-7.75	1.75	8.00	7.0-8.5	1.50	5.00	3.0-7.5	4.50	4.00	3.0-5.75	2.75	8.00	7.0-8.5	1.50
Self-management and self-direction	7.00	6.0-8.5	2.50	7.50	6.0-8.75	2.75	7.00	5.5-8.5	3.00	8.00	6.5-9.0	2.50	8.00	6.25-9.0	2.75	8.00	6.0-8.5	2.50
Learning ability	7.00	6.0-8.5	2.50	7.00	6.25-8.75	2.50	6.00	5.5-8.5	3.00	8.00	7.0-8.0	1.00	8.00	7.0-8.0	1.00	7.00	6.0-8.5	2.50
Social network	7.00	7.0-8.0	1.00	7.00	7.0-8.0	1.00	7.00	4.5-8.5	4.00	8.00	7.0-8.5	1.50	8.00	7.0-9.0	2.00	7.00	4.5-8.0	3.50
Need for technical nursing care	6.00	5.5-8.0	2.50	6.00	5.0-8.0	3.00	7.00	6.0-8.5	2.50	7.00	6.0-8.0	2.00	7.50	6.0-8.75	2.75	6.00	4.5-8.0	3.50
