# Supplementary material for: Identifying client characteristics to predict homecare use more accurately: a Delphi-study involving nurses and homecare purchasing specialists
Source: BMC Health Serv Res. 2022 Mar 25;22:394. doi: 10.1186/s12913-022-07733-9 (PMC8957197; doi:10.1186/s12913-022-07733-9)
Supplement: Supplementary file 2 [file 12913_2022_7733_MOESM2_ESM.docx]

Additional file 2. The 53 unique client characteristics that were suggested

The 53 suggested client characteristics (per category of predictors) that resulted from grouping the 142 client characteristics suggested by the participants:

- Category 'Daily functioning' (11):
  1. Daily routine
  2. Use of telephone
  3. Housework
  4. Grocery shopping
  5. Sleeping
  6. Managing (financial) administration
  7. Taking the initiative
  8. ADL tasks*
  9. Stocking*
  10. Changing incontinence material*
  11. Medication*
- Category 'Physical health status' (13):
  12. Multimorbidity**
  13. Diabetes
  14. Skin problems**
  15. Airway functioning
  16. Vision and hearing**
  17. Pain
  18. Polypharmacy
  19. Risk of falls
  20. Physical fitness
  21. Malnutrition**
  22. Excess weight
  23. Mobility*
  24. Progressive disease*
- Category 'Mental health status and behaviour' (7):
  25. Mental functioning**
  26. Resistance to receiving care
  27. Resilience**
  28. Dementia**
  29. Communication skills
  30. Self-management and self-direction**
  31. Cognitive skills*
- Category 'Health literacy' (7):
  32. Health literacy
  33. Healthy lifestyle
  34. Learning ability**
  35. Compliance with therapy
  36. Digital skills
  37. Awareness of support options
  38. Capacity for self-care*
- Category 'Social environment and network' (4):
  39. Loneliness
  40. Social network**
  41. Participation in social activities
  42. Meaning
- Category 'Other' (11):
  43. Financial resources
  44. Formal care network
  45. Living in an urban area
  46. Need for technical nursing care**
  47. Quality of transfer from the hospital
  48. Quality of life
  49. Frailty
  50. Availability of facilities
  51. Care needs at multiple levels*
  52. Terminal status*
  53. (Illness) prognosis/Stability*

* Client characteristics that (partially) overlap with one or several of the 11 pre-existing client characteristics that were already included in the Case-Mix Short Form questionnaire.
** The 11 suggested client characteristics that were selected by the researchers for further assessment.
