# Supplementary material for: Identifying client characteristics to predict homecare use more accurately: a Delphi-study involving nurses and homecare purchasing specialists
Source: BMC Health Serv Res. 2022 Mar 25;22:394. doi: 10.1186/s12913-022-07733-9 (PMC8957197; doi:10.1186/s12913-022-07733-9)
Supplement: Supplementary file 3 [file 12913_2022_7733_MOESM3_ESM.docx]

Additional file 3. Sensitivity analysis regarding the relevance of client characteristics (median and IQR) per Delphi-round, comparing results for all participants, nurses, and insurers

	Delphi round 1									Delphi round 2								
	Total			Nurses			Insurers			Total			Nurses			Insurers		
	Median	Q1-Q3	IQR	Median	Q1-Q3	IQR	Median	Q1-Q3	IQR	Median	Q1-Q3	IQR	Median	Q1-Q3	IQR	Median	Q1-Q3	IQR
Pre-existing																		
Meal preparation	6.00	2.75-7.0	4.25	6.00	5.0-7.0	2.00	4.00	1.75-6.25	4.50	5.00	2.5-7.0	4.50	6.00	4.25-7.0	2.75	3.00	2.0-4.0	2.00
Eating and drinking	7.00	5.75-8.0	2.25	7.00	6.0-8.75	2.75	6.00	4.75-8.0	3.25	7.00	5.0-7.0	2.00	7.00	7.0-7.75	0.75	5.00	3.5-6.0	2.50
Continence	6.00	4.5-7.0	2.50	6.00	5.0-7.0	2.00	5.50	3.0-6.5	3.50	5.00	3.0-6.5	3.50	5.50	3.25-7.0	3.75	5.00	3.0-5.5	2.50
Toileting	7.00	3.75-8.25	4.50	7.00	3.75-9.0	5.25	4.50	3.75-7.25	3.50	7.00	4.0-8.0	4.00	7.50	5.25-8.0	2.75	4.00	3.5-7.5	4.00
Mobility	7.00	5.0-9.0	4.00	7.50	5.25-9.0	3.75	6.50	4.75-7.5	2.75	7.00	5.0-7.5	2.50	7.00	5.0-8.0	3.00	5.00	4.5-7.0	2.50
Dressing	7.00	5.0-8.0	3.00	6.50	5.0-8.0	3.00	7.00	5.0-8.25	3.25	6.00	5.0-7.5	2.50	6.50	5.0-7.75	2.75	5.00	5.0-7.5	2.50
Washing	7.00	5.0-7.0	2.00	7.00	5.0-7.0	2.00	7.00	5.75-7.5	1.75	-	-	-	-	-	-	-	-	-
Medication use	7.00	4.75-8.0	3.25	7.50	6.25-8.75	2.50	4.50	2.75-7.25	4.50	7.00	5.0-8.0	3.00	7.00	6.25-8.75	2.50	5.00	3.5-6.0	2.50
Cognitive skills for daily decision making	8.00	7.0-9.0	2.00	8.50	7.25-9.0	1.75	7.00	3.75-9.0	5.25	-	-	-	-	-	-	-	-	-
Informal care	8.00	6.0-9.0	3.00	8.00	7.25-9.0	1.75	7.50	5.75-9.0	3.25	9.00	6.5-9.0	2.50	9.00	8.25-9.0	0.75	7.00	5.5-9.0	3.50
Illness prognosis	8.00	7.0-9.0	2.00	8.00	7.0-9.0	2.00	8.00	6.0-8.25	2.25	-	-	-	-	-	-	-	-	-
Suggested																		
Multimobidity	7.00	6.5-7.5	1.00	7.00	7.0-7.75	0.75	7.00	6.0-7.5	1.50	7.00	7.0-7.0	0.00	7.00	7.0-7.75	0.75	7.00	5.5-7.0	1.50
Skin problems	7.00	5.0-8.0	3.00	7.00	5.0-8.0	3.00	7.00	4.0-8.0	4.00	7.00	5.0-8.0	3.00	7.00	5.0-8.0	3.00	5.00	4.0-7.0	3.00
Vision and hearing	5.00	3.5-6.0	2.50	6.00	4.25-6.0	1.75	4.00	3.0-5.5	2.50	5.00	3.0-6.0	3.00	5.50	3.25-6.0	2.75	3.00	3.0-4.5	1.50
Malnutrition	6.00	4.5-6.0	1.50	6.00	4.25-6.75	2.50	5.00	4.5-5.5	1.00	6.00	5.0-6.5	1.50	6.00	5.25-7.75	2.50	5.00	3.5-5.0	1.50
Mental functioning	7.00	6.0-8.0	2.00	7.00	6.0-8.0	2.00	7.00	6.0-7.5	1.50	7.00	6.0-8.0	2.00	7.00	6.25-8.0	1.75	7.00	6.0-7.5	1.50
Resilience	7.00	6.5-7.5	1.00	7.00	6.25-8.0	1.75	7.00	6.5-7.0	0.50	7.00	6.5-8.0	1.50	8.00	6.25-8.0	1.75	7.00	6.5-7.5	1.00
Dementia	7.00	6.5-8.0	1.50	7.00	6.0-7.75	1.75	8.00	7.0-8.5	1.50	5.00	3.0-7.5	4.50	4.00	3.0-5.75	2.75	8.00	7.0-8.5	1.50
Self-management and self-direction	7.00	6.0-8.5	2.50	7.50	6.0-8.75	2.75	7.00	5.5-8.5	3.00	8.00	6.5-9.0	2.50	8.00	6.25-9.0	2.75	8.00	6.0-8.5	2.50
Learning ability	7.00	6.0-8.5	2.50	7.00	6.25-8.75	2.50	6.00	5.5-8.5	3.00	8.00	7.0-8.0	1.00	8.00	7.0-8.0	1.00	7.00	6.0-8.5	2.50
Social network	7.00	7.0-8.0	1.00	7.00	7.0-8.0	1.00	7.00	4.5-8.5	4.00	8.00	7.0-8.5	1.50	8.00	7.0-9.0	2.00	7.00	4.5-8.0	3.50
Need for technical nursing care	6.00	5.5-8.0	2.50	6.00	5.0-8.0	3.00	7.00	6.0-8.5	2.50	7.00	6.0-8.0	2.00	7.50	6.0-8.75	2.75	6.00	4.5-8.0	3.50
